# Classification, detection, and segmentation performance of image-based AI in intracranial aneurysm: a systematic review

**DOI:** 10.1186/s12880-024-01347-9

**Published:** 2024-07-02

**Authors:** Zhiyue Zhou, Yuxuan Jin, Haili Ye, Xiaoqing Zhang, Jiang Liu, Wenyong Zhang

**Affiliations:** 1 School of Medicine, Southern University of Science and Technology, Southern University of Science and Technology, Shenzhen, China; 2 Research Institute of Trustworthy Autonomous Systems and Department of Computer Science and Engineering, Southern University of Science and Technology, Shenzhen, China; 3 School of Ophthalmology and Optometry and Eye Hospital, Wenzhou Medical University, Wenzhou, China

**Keywords:** Intracranial aneurysm, Artificial intelligence, Medical imaging

## Abstract

**Background:**

The detection and management of intracranial aneurysms (IAs) are vital to prevent life-threatening complications like subarachnoid hemorrhage (SAH). Artificial Intelligence (AI) can analyze medical images, like CTA or MRA, spotting nuances possibly overlooked by humans. Early detection facilitates timely interventions and improved outcomes. Moreover, AI algorithms offer quantitative data on aneurysm attributes, aiding in long-term monitoring and assessing rupture risks.

**Methods:**

We screened four databases (PubMed, Web of Science, IEEE and Scopus) for studies using artificial intelligence algorithms to identify IA. Based on algorithmic methodologies, we categorized them into classification, segmentation, detection and combined, and then their merits and shortcomings are compared. Subsequently, we elucidate potential challenges that contemporary algorithms might encounter within real-world clinical diagnostic contexts. Then we outline prospective research trajectories and underscore key concerns in this evolving field.

**Results:**

Forty-seven studies of IA recognition based on AI were included based on search and screening criteria. The retrospective results represent that current studies can identify IA in different modal images and predict their risk of rupture and blockage. In clinical diagnosis, AI can effectively improve the diagnostic accuracy of IA and reduce missed detection and false positives.

**Conclusions:**

The AI algorithm can detect unobtrusive IA more accurately in communicating arteries and cavernous sinus arteries to avoid further expansion. In addition, analyzing aneurysm rupture and blockage before and after surgery can help doctors plan treatment and reduce the uncertainties in the treatment process.

## Introduction

**Fig. 1:**
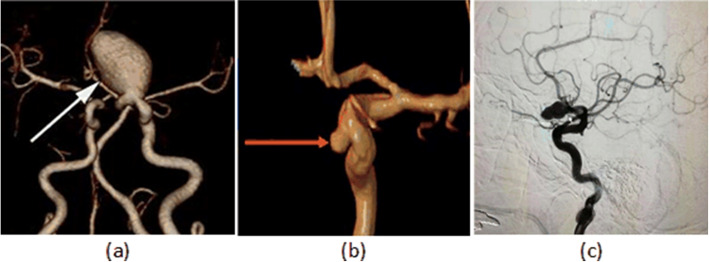
Clinical Imaging Examples of IAs. **a** CTA Example; **b** MRA Example; **c** DSA Example

Intracranial aneurysms (IAs) are widely recognized as both common and potentially lethal conditions [1, 2]. Ruptured intracranial aneurysms are the primary cause of spontaneous subarachnoid hemorrhage, which can result in severe consequences such as cerebral vasospasm, hydrocephalus and hyponatremia [3]. Therefore, early detection of intracranial aneurysms is particularly crucial. Medical imaging techniques play a significant role in the detection, risk assessment, treatment, and prognosis of intracranial aneurysms. Key imaging techniques used include computed tomography angiography (CTA) [4, 5], magnetic resonance angiography (MRA) [6, 7], digital subtraction angiography (DSA) [8, 9], and the examples are shown in Fig. 1.

CTA has high sensitivity and specificity in diagnosing intracranial aneurysms. However, due to the almost equal absorption effect of contrast agents and calcium on ionizing radiation, CTA is unsuitable for detecting small IAs near the skull [10]. MRA is a non-invasive examination that can accurately measure the size of aneurysms and their positional relationship with the parent artery. However, MRA has drawbacks, such as long imaging time and low resolution [11]. DSA has the advantages of high spatial resolution and has long been used as the gold standard for diagnosing intracranial aneurysms, but invasive procedures increase the risk of complications [12]. Consequently, the application of these imaging techniques generally depends on diverse circumstances and requirements of patients. Traditionally, these medical images are analyzed and interpreted by experienced radiologists, whose expertise is essential for accurate diagnosis [13]. Nevertheless, manually analyzing medical images can be a laborious process, prone to inaccuracies, and may vary depending on the observer.

Considering the exceptional efficacy of artificial intelligence (AI) in image-related tasks, methods utilizing traditional machine learning (ML) and deep learning (DL) have recently emerged for the recognition of intracranial aneurysms [14]. Artificial intelligence can provide precise analysis of the morphology, size, and location of intracranial aneurysms, offering clinicians more comprehensive information to better formulate diagnosis and treatment plans. Currently, AI-based classification, detection, segmentation, and composite models for intracranial aneurysms have been developed, aiding in the assessment of rupture risk and prognostic prediction for intracranial aneurysms [15, 16]. In general, AI models trained on extensively annotated data can effectively and automatically localize suspected IA regions within images, thereby enhancing diagnostic efficiency and accuracy.

To date, little meta-analysis focuses specifically on the performance of image-based AI in the diagnosis of intracranial aneurysms. Our study aims to conduct a comprehensive review of AI-based IA recognition methods, and systematically evaluate their performance across various IA recognition tasks in medical imaging, providing a thorough overview of the current state of the field.

## Methods

### Search strategy

A systematic literature review of PubMed, Web of Science, IEEE and the Scopus Library was manually conducted from their establishment date until March 2024 to identify relevant reports. The databases were searched using the terms[(intracranial aneurysm classification), (intracranial aneurysm segmentation), (intracranial aneurysm detection) and (intracranial aneurysm recognition)]. The search strategies incorporated the Medical Subject Headings terms and keywords. References lists of the relevant articles were also screened.

### Study selection

All collected studies were screened for eligibility by two independent reviewers. This review focuses on regional detection and localization recognition of IA. We have filtered out studies that do not align with the topic of this article, including aneurysm surgery and morphological analysis. Furthermore, we divide our research work into three types based on algorithmic forms: image segmentation, classification, and detection. Therefore, we screened the preliminary search results based on the following criteria: (1) original articles published until March 2024; (2) provide only English; (3) the research content includes aneurysm detection and recognition, and the method categories can be summarized into one of three types of algorithms; (4) only human subjects are involved. Any differences between the authors reviewers were resolved through consensus in the presence of a third reviewer.

### Study selection

The first stage of the screening method involves evaluating the titles and abstracts of the identified papers, removing those unrelated to the research topic, and then eliminating duplicate papers. Subsequently, full texts of relevant articles are retrieved for final evaluation, followed by an assessment based on eligibility criteria. The remaining articles are then thoroughly reviewed to extract the required data. This screening process was conducted by two researchers (Z.Z and Y.J). In cases of disagreement, they consulted with a third researcher (H.Y) to discuss article selection, remove irrelevant/unqualified papers, and extract data. The collected articles were utilized to extract the following variables: first author, year of publication, study type, sampling method, annotation method, sample size, accuracy, sensitivity, and specificity.

**Fig. 2:**
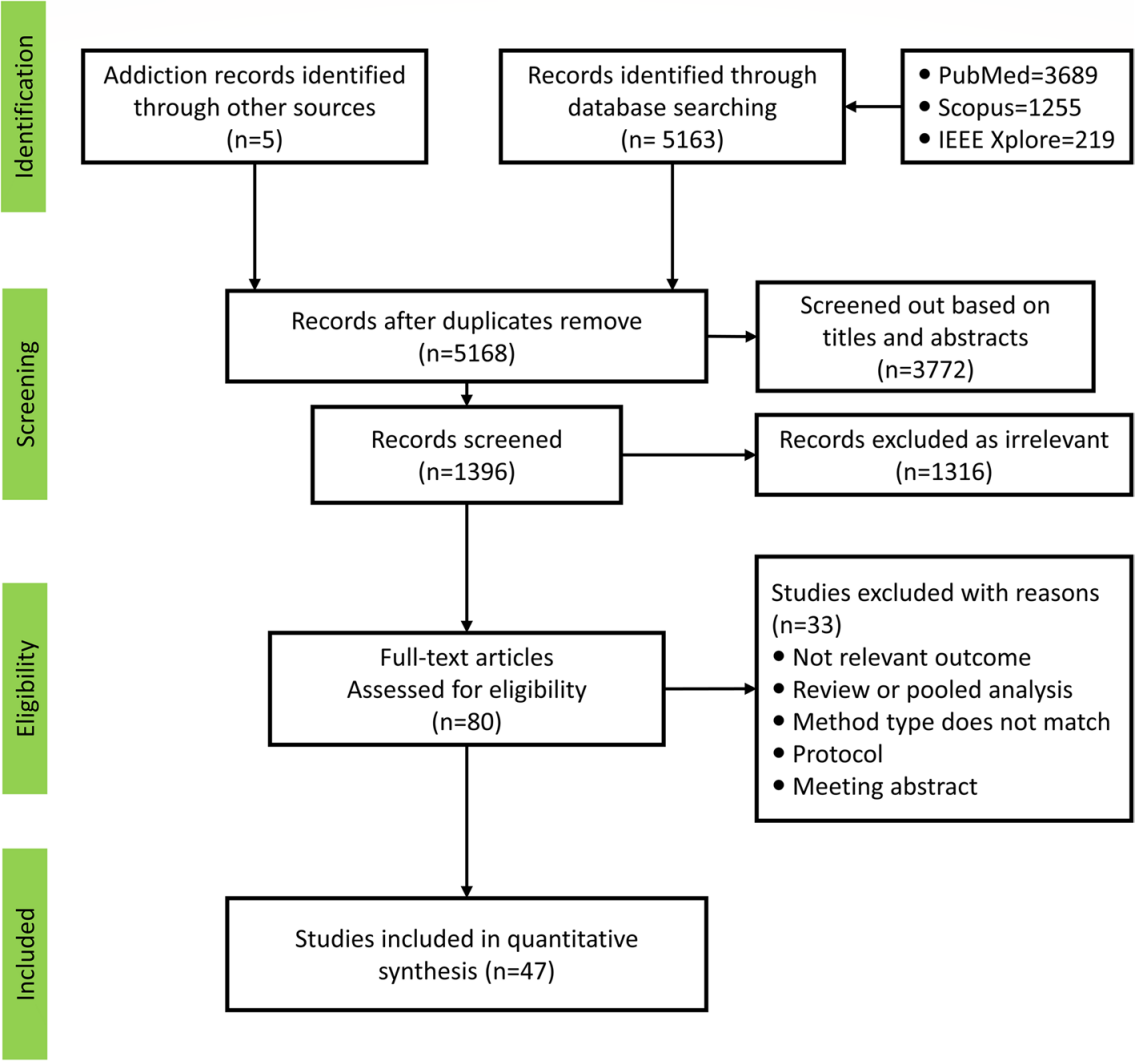
The flow of searching and selecting articles

## Result

### Study results

In the first stage of the search process, a total of 5168 papers were identified; 3772 were eliminated due to irrelevance. After removing duplicate entries, 1396 remained. Papers that did not meet eligibility criteria or align with the current research objectives were disqualified in the subsequent stage. Following the application of eligibility criteria, 80 articles were excluded. Finally, 47 articles were retained for analysis. The article selection process is described in detail as shown in Fig. 2.

### Methodologies overview

**Classification-based structure:** As shown in Table 1, based on classification models, the recognition of IAs aims to determine whether IAs exist in the image or to assess the status of IAs (such as potential blockage or rupture), mainly divided into two categories:

(1) Based on morphological features [17–21]: by extracting morphological features of intracranial aneurysm images, such as size, shape and edges, and then using traditional machine learning methods or rule-based methods for classification. This method relies on manually designed feature extraction and classification algorithms, requires high demands on the morphological features of intracranial aneurysms, and may not capture subtle variations in the images.

(2) Based on imaging features [22–24, 4, 9, 25–35]: using deep learning techniques, such as convolutional neural networks (CNNs), to learn feature representations from intracranial aneurysm images and apply them to classification tasks. This method does not require manual feature extraction but learns feature representations from raw image data through end-to-end learning, which can better capture the complex features of intracranial aneurysms and improve classification performance.

Overall, classification methods based on morphological features rely heavily on the accuracy of upstream feature extraction, and the model’s performance is sensitive to changes in imaging quality and feature extraction thresholds. Samples with significant imaging differences often fail to exhibit robust inference performance. In contrast, classification methods based on imaging features can achieve end-to-end recognition of IA, and through training on large-scale datasets, they can robustly adapt to imaging variations. However, the automatically extracted image features lack a certain level of clinical interpretability.

**Detection-based structure:** As shown in Table 2 [36–39], object detection aims to identify the categories and bounding boxes of specific objects in images [40]. In the task of IA recognition, the purpose is to mark the area where IAs are located by bounding boxes, allowing doctors to quickly locate potential IA regions in the image. Specifically, the processing pipeline of object detection models includes feature extraction, region regression, and category prediction. In the feature extraction stage, the model extracts feature representations from the image, typically using CNNs to capture image features. Next, in the region regression stage, these features are utilized to predict the position and size of the bounding boxes, accurately locating the target objects. Finally, in the category prediction stage, these features are used to determine the category of each object within the bounding box, thereby completing the object detection task.

Compared to classification models, object detection not only provides more detailed information but also offers spatial location and category of objects. However, traditional object detection methods cannot segment the specific boundaries of IAs. It requires incorporating a segmentation module at the end of the model to segment the foreground mask of each object within the bounding box, known as instance segmentation. However, similar to segmentation methods, its prediction results also suffer from false positives/false negatives, necessitating further calibration by doctors to obtain the final diagnosis.

**Table 1:** A summary of intracranial aneurysm recognition using image classification methodology

Author/Year	Model	Imaging	Total/Intervention	Training/Test	Accuracy %	Sensitivity %	Specificity %
			Sample size	Sample size			
Paliwal et al. 2018 [17]	MLP	DSA	80/84	64/20	90.0	-	-
Park et al. 2019 [22]	3D CNN	CTA	726/282	611/115	89.3	83.1	96.0
Ueda et al. 2019 [23]	3D CNN	MRA	750/828	682/67	90.0	87.0	95.0
Joo et al. 2019 [24]	3D CNN	MRA	574/614	468/106	-	85.7	98.0
Chen et al. 2020 [18]	MLP	CTA	915/915	807/108	80.0	81.6	73.5
Detmer et al. 2020 [19]	MLP	DSA	1264/1880	1061/203	78.6	48.4	89.5
Zeng et al. 2020 [25]	SIF 2D CNN	DSA	300/263	150/150	98.8	99.3	98.1
Bhurwani et al. 2020 [20]	MLP	DSA	190/126	135/55	77.9	92.0	57.0
Zhu et al. 2020 [21]	MLP	DSA	1897/2067	1656/411	82.4	51.5	92.9
Yang et al. 2021 [26]	2D CNN	CTA	1068/1337	534/534	-	91.9	90.9
Joo et al. 2021 [27]	3D CNN	MRA	800/603	468/322	-	91.1	93.9
Wei et al. 2022 [28]	3D CNN	CTA	212/224	170/42	74.5	84.9	18.2
Li et al. 2023 [29]	3D CNN	CTA	556/731	400/331	93.0	-	-
Xie et al. 2023 [4]	3D CNN	CTA	106/106	71/35	89.8	-	-
Niemann et al. 2023 [30]	Surface Meshes Model	DSA	84/84	60/24	83.3	-	-
Hu et al. 2023 [9]	SR Method	DSA	263/263	187/76	96.1	94.4	97.5
Wang et al. 2023 [31]	3D CNN	DSA	110/114	75/35	-	82.9	-
Timmins et al. 2023 [32]	Surface Meshes Model	MRA CTA	235/235	93/142	-	52.0	-
			20/20	0/20		48.3	
Noto et al. 2023 [33]	3D CNN	MRA	246/246	197/49	-	83.0	-
Peng et al. 2024 [34]	2D CNN	CTA	101/101	87/14	-	AUC=93.2	
Cao et al. 2024 [35]	3D PCC Model	DSA	623/623	464/159	82.0	72.0	86.0

*MLP* Multilayer Perceptron, *SIF* Spatial Information Fusion, *SR* Sparse Representation, *PCC* Point Cloud Classification, *CNN* Convolutional Neural Network, *AUC* Area Under Curve

**Table 2:** A summary of intracranial aneurysm recognition using object detection methodology

Author/Model	Model	Imaging	Total/Intervention	Training/Test	Sensitivity %	Specificity %	MIoU %
			Sample size	Sample size			
Dai et al. 2019 [36]	Faster -RCNN	CTA	311/344	208/103	94.3	-	55.0
Liao et al. 2019 [37]	2D LSTM	DSA	500/500	400/100	88.0	83.0	-
Duan et al. 2019 [38]	Two-stage 2D CNN	DSA	261/261	241/40	93.5	96.0	91.0
Assis et al. 2023 [39]	Pose Estimation Model	MRA	132/132	106/26	82.9	-	-

*LSTM* Long Short-term Memory Network, *MIoU* Mean Intersection over Union

**Table 3:** A summary of intracranial aneurysm recognition using semantic segmentation methodology

Author/Model	Model	Imaging	Total/Intervention	Training/Test	PA %	Dice %	IoU %
			Sample size	Sample size			
Podgorsak et al. 2023 [41]	2D UNet	DSA	350/313	250/100	-	90.3	82.3
Jin et al. 2020 [42]	2D UNet	DSA	395/977	249/146	-	53.3	-
Yuan et al. 2022 [43]	3D UNet	MRA	93/113	70/23	-	74.6	-
Liu et al. 2022 [44]	3D PCS Model	MRA	116/116	93/23	-	95.5	91.5
Liu et al. 2023 [45]	3D PCS Model	MRA	116/116	93/23	95.2	95.5	91.4
Claux et al. 2023 [46]	3D UNet	MRA	49/63	24/25	-	78.0	-
Ham et al. 2023 [47]	3D UNet	MRA	135/135	120/15	77.2	22.6	-
Mu et al. 2023 [48]	3D UNet	DSA	23/23	15/8	-	86.8	-
Zhang et al. 2023 [49]	3D UNet	DSA	300/300	240/60	-	91.1	85.9
Abdullah et al. 2023 [50]	2D UNet	DSA	409/436	327/82	99.9	-	96.8
Guo et al. 2024 [51]	SCTR Model	MRA	113/125	90/23	-	55.9	-
Estrella-Ibarra et al. 2024 [52]	3D PCS Model	MRA	116/116	93/23	-	-	89.8

*PCS* Point Cloud Segmentation, *SCTR* Staged Cluster Transformers, *PA* Pixel Accuracy, *MIoU* Mean Intersection over Union

**Table 4:** A summary of intracranial aneurysm recognition using hybrid structure methodology

Author/Model	Model	Imaging	Total/Intervention	Training/Test	Performance (%)
			Sample size	Sample size	
Shi et al. 2020 [16]	3D UNet + 3D CNN	CTA	1077/838	927/150	**Classification**
					Acc: 86.0 Sen: 97.3 Spe:74.7
					**Segmentation**
					Dice: 75.0
You et al. 2022 [5]	3D UNet + 3D UNet	CTA	2272/2938	1606/352	**Classification**
					Recall: 96.4
					**Segmentation**
					Dice: 78.3
Wu et al. 2022 [55]	3D UNet + 3D CNN	CTA	1508/1710	1205/303	**Classification**
					Sen: 76.0 Spe:80.0
					**Segmentation**
					Dice: 85.4
Yang et al. 2020 [56]	3D PCS + PCC Model	MRA	2025/215	1620/405	**Classification**
					F1-Score: 89.2
			116/116	93/23	**Segmentation**
					Dice: 87.9 MIoU: 80.1
Timmins et al. 2021 [57]	2D UNet + 2D CNN	MRA	254/282	113/141	**Classification**
					Sen: 70.0
					**Segmentation**
					Dice: 63.0
Shao et al. 2022 [58]	3D PCS + PCC Model	MRA	2025/215	1620/405	**Classification**
					Acc: 86.6 F1-Score: 82.9
			116/116	93/23	**Segmentation**
					MIoU: 48.6
Ou et al. 2022 [59]	3D UNet + 3D CNN	DSA	131/157	109/22	**Classification**
					Acc: 94.4 Sen: 85.8
					**Segmentation**
					Dice: 83.6 MIoU: 73.8
Irfan et al. 2023 [60]	2D UNet + 2D CNN	DSA	408/369	267/141	**Classification**
					Acc: 79.2 F1-Score: 71.4
					**Segmentation**
					PA: 92.7 Dice: 74.0 MIoU: 78.0
Nageler et al. 2023 [61]	3D UNet + 3D CNN	CTA	121/121	100/21	**Classification**
					Acc: 85.0
					**Segmentation**
					Dice: 94.4
Cao et al. 2024 [62]	3D PCS + PCC Model	MRA	2025/215	1620/405	**Classification**
					F1-Score: 94.4
			116/116	93/23	**Segmentation**
					Dice: 91.4 MIoU: 86.0

*CNN* Convolutional Neural Network, *PCC* Point Cloud Classification, *PCS* Point Cloud Segmentation

**Segmentation-based structure:** As shown in Table 3, [41–52], the goal of semantic segmentation is to classify each pixel in the image into a specific category to achieve pixel-level classification [53]. Segmentation-based IA recognition aims to accurately segment the specific boundaries of IAs in images. A common semantic segmentation model adopts a U-shaped architecture [54], which includes both an encoder and a decoder. Specifically, the encoder is responsible for extracting high-level semantic information from input images, typically composed of convolutional layers and pooling layers. This process gradually reduces the image size while increasing the depth of feature maps. In contrast, the decoder is tasked with mapping the features extracted by the encoder back to the original image size and generating pixel-level prediction results. Typically, the decoder consists of transposed convolutional layers and upsampling layers, which gradually restore the image size while preserving semantic information.

Additionally, segmentation methods offer greater interpretability compared to classification methods as they can clearly delineate the boundaries of IAs. However, since semantic segmentation identifies regions belonging to IAs as the same category when faced with the situation of multiple aneurysms adhering together, they will be recognized as a unified whole, making it unable to accurately distinguish the specific boundaries of different IA instances.

**Hybrid-based structure:** As shown in Table 4, [16, 5, 55–62] The hybrid-based IA recognition model adopts a two-step approach to accurately identify and classify intracranial aneurysms. Firstly, a segmentation model is utilized to delineate the specific boundaries of IAs. This segmentation process enables precise localization of the aneurysm regions within the image. Subsequently, the segmented regions are input into a classification model, which determines the category of each IA, such as the risk of rupture or obstruction. By combining segmentation and classification techniques, hybrid-based models leverage the strengths of both approaches, allowing for comprehensive analysis and accurate diagnosis of IAs. This hybrid approach yields more detailed recognition results incrementally, but it also faces challenges similar to other domains’ hybrid models, such as high computational complexity and issues with error propagation.

### Evaluation of IA recognition based on classification

A total of 21 studies implemented IA recognition based on classification models [4, 9, 17–35], as shown in Table 1. Combined with Tables 2, 3, and 4, it can be seen that classification is currently the predominant method for IA recognition. Classification studies report average accuracy, sensitivity, and specificity as outcome measures, with the patient cohort size ranging from 20 to 1897. Five studies [17–21] utilized pattern classification based on morphological features, focusing mainly on identifying aneurysm states such as rupture and obstruction risk prediction. These studies are primarily applied in postoperative follow-up, where aneurysm appearance parameters and patient clinical features are extracted to form feature vectors, which are then inputted into multilayer perceptron (MLP) to predict the aneurysm’s trend in status. The test group’s average accuracy ranges from 77.9% to 90.0%. Specifically, this morphology-based classification pattern can effectively infer the aneurysm’s status in postoperative follow-up diagnosis, providing valuable auxiliary diagnostic reference results.

In addition, 16 studies [22–29, 4, 30, 9, 31–35] utilized a pattern classification based on image features, aiming to extract deep semantic features from images using deep learning models and utilize these features for automatic classification and recognition of IAs. The test group’s average accuracy ranged from 74.5% to 98.8%, with average sensitivity ranging from 48.3% to 99.3% and average specificity ranging from 18.2% to 98.1%. Overall, DL models showed relatively consistent performance in IA image classification tasks in terms of accuracy, but there were significant differences in specificity and sensitivity performance, mainly influenced by factors such as training data size, imaging sampling, and the proportion of positive samples.

### Evaluation of IA recognition based on object detection

Four studies [36–39] conducted IA recognition based on object detection, as shown in Table 2. Detection studies reported average sensitivity, specificity, and Mean Intersection over Union (MIoU) as outcome measures, with the patient cohort size ranging from 132 to 500. These detection models first locate and label the IA regions in the images, and then further classify and identify each region. The test group’s average sensitivity ranged from 82.9% to 94.3%, average specificity ranged from 83.0% to 96.0%, and MIoU ranged from 55.0% to 91.0%. However, compared to classification-based models, object detection models require more computational resources and time to process each image, thus may have some limitations in practical applications. Additionally, while the bounding boxes outputted by object detection models can locate the position and rough boundaries of IAs, they cannot be further utilized for calculating clinical parameters such as aneurysm area, maximum diameter ratio and roundness, unlike the boundary masks outputted by segmentation models. In real clinical diagnosis, object detection-based methods can assist doctors in locating suspected IA regions in images, but subsequent quantification and analysis of clinical parameters still rely on manual annotation by doctors and semi-automatic measurement tools. Therefore, it may be necessary to adapt it to the actual needs of clinical diagnosis by extending it into a model structure for instance segmentation.

### Evaluation of IA recognition based on semantic segmentation

Twelve studies [41–52] conducted IA recognition based on semantic segmentation, as shown in Table 3. The detection studies reported average Pixel Accuracy (PA), Dice coefficient, and MIoU as outcome measures, with the patient group size ranging from 23 to 409. These segmentation models first extracted high-level semantic information from input images through an encoder. Then, through a decoder, they mapped the features extracted by the encoder back to the original image size, generating pixel-level prediction results. The test group’s average PA ranged from 77.2% to 99.9%, average Dice ranged from 22.6% to 95.5%, and MIoU ranged from 82.3% to 96.8%. From Table 3, it can be observed that while the models performed well in PA, the performance of Dice was relatively lower. This could be due to the overall low area of IAs in the images, allowing the model to achieve high PA even if it predicts all regions as negative areas. Conversely, Dice and MIoU simultaneously evaluate the overlap of foreground and background regions, thus better reflecting the model’s accuracy and coverage in IA boundary segmentation.

Furthermore, most segmentation models adopted the UNet [54] design structure, incorporating skip connections between the encoder and decoder. This helps the model capture different levels of feature information more effectively and alleviates the issue of information loss. Skip connections allow the decoder to utilize lower-level feature information from the encoder, thereby better recovering image details and boundary information. The adoption of this structure contributes to improving the accuracy and robustness of the model in IA boundary delineation. Additionally, Guo et al. [51] introduced an encoder based on the Transformer structure, utilizing its self-attention mechanism to enhance the model’s ability to model global and local features, further improving performance. Overall, semantic segmentation-based models have made significant progress in IA recognition, providing important diagnostic information to clinicians through pixel-level prediction, and accurately delineating IA boundaries.

### Evaluation of IA recognition based on hybrid model

Ten studies [16, 5, 55–62] employed hybrid models for IA recognition, as shown in Table 4. It is evident that these 10 studies all adopted a two-stage hybrid structure based on segmentation and classification. Consequently, the hybrid studies reported measurements for both segmentation and classification, including average accuracy, average specificity, and average sensitivity for classification, and Dice coefficient, MIoU, and F1-Score for segmentation. The patient cohort sizes ranged from 116 to 2272. The test groups showed average accuracy ranging from 79.2% to 94.4%, average sensitivity from 52.0% to 97.3%, average Dice coefficient from 74.0% to 94.4%, MIoU from 48.6% to 86.0%, and average F1-Score from 71.4% to 94.4%.

Moreover, a significant contribution introduced a 3D point cloud segmentation and classification dataset for IAs named IntrA dataset [56]. This dataset reconstructed 2D MRA scan images into 3D point cloud data format and meticulously annotated healthy vascular segments and aneurysmal segments. This innovative approach brought the IA recognition task into the realm of 3D point clouds, offering a fresh perspective for recognition. Subsequently, studies [44, 45, 52, 56, 58, 62] conducted model structure innovations and performance evaluations on this dataset, driving further advancements in IA recognition technology. It can be observed that while models based on hybrid structures may entail higher complexity, the two-stage models can balance both the accuracy and completeness of output results.

## Discussion

### Potential technical challenges and prospects

Our research indicates that AI is highly effective in the evaluation of IA, enabling the efficient analysis of extensive imaging datasets and fostering standardized assessments that reduce diagnostic discrepancies among clinicians. However, challenges persist, such as varying training sample sizes in different studies and the high computational demands of complex models. To achieve better clinical applications, further research could be conducted in the following aspects:

1. **Reducing the latency of AI systems**: Fully functional AI systems are often built on composite models, leading to delays in system recognitions, which are not conducive to real-time applications such as surgical treatments. Therefore, reducing the latency of AI system inference to meet clinical diagnostic needs is necessary. This could involve optimizing model structures to reduce computational complexity or introducing efficient inference algorithms to improve system response time.
2. **Increasing the interpretability of reasoning**: Current AI algorithms can detect subtle intracranial aneurysms in both arterial and venous sinuses, but lack interpretability in assessing postoperative recurrence risks or occlusion probabilities [63]. Comprehensive postoperative evaluations require consideration of patients’ medical histories and health records in addition to their imaging data. Therefore, AI models for postoperative assessment may need to incorporate patients’ electronic health records (EHR) and image features to provide interpretability for the reliability of diagnostic results while outputting auxiliary diagnostic outcomes.
3. **Coordinating the diagnosis of multiple neurological disorders**: In neurological disorders, IAs may coexist with other conditions such as atherosclerosis and thrombosis. In real clinical diagnostic scenarios, there is a greater need to consider multi-task, multi-modal situations. Therefore, comprehensive diagnosis of neurological disorders requires joint analysis of patients’ multi-modal images. Additionally, it may be necessary to establish links between different lesions through knowledge graph construction [64]. Consequently, designing artificial intelligence systems that cover a wide range of lesions with high sensitivity still faces many technical challenges and requires clinical validation.
4. **Introducing large-language pretraining models**: Influenced by the research trend of large-language models (LLM) [65], how to improve the recognition accuracy of specific tasks with the help of the prior weights of LLM is one of the current research hotspots. Due to the high cost of clinical data acquisition in IA, de novo training models tend to be less effective with limited datasets. Therefore, it is an idea worth exploring in the future to achieve accurate recognition within the model by leveraging the prior knowledge of the large model, even when confronted with limited IA data.
5. **Promoting open data sharing**: Currently, most studies rely on proprietary datasets, leading to inconsistencies in algorithm design and comparison. Additionally, there are few open-source datasets for IAs recognition, while high-quality public datasets are crucial as they enhance the credibility and replicability of research findings. For instance, the TCGA dataset [66] is utilized in pathological image analysis, and the CheXpert dataset [67] in lung X-ray studies. Moreover, integrating multiple datasets from various sources can facilitate cross-domain or multitask learning research, thereby enriching the scope and applicability of work in this field. Therefore, it is vital to expand the availability of open datasets for IA recognition and to establish clear performance evaluation criteria, which are key focuses for future research in this area.

### Strengths

With the increasing popularity of AI and computer vision methods in medical image analysis and the auxiliary diagnosis of IAs, more and more studies have discussed the feasibility of automatic IA recognition. However, we have yet to find any reviews that provide a detailed methodological division and performance evaluation for IA recognition based on AI models.

In this comprehensive review, we systematically categorize existing studies on IAs recognition facilitated by AI, dividing them into four methodological groups: classification, detection, segmentation, and hybrid approaches. This structured classification aids readers and researchers in discerning the methodological distinctions across studies. Our review draws from an extensive selection of studies found in leading scientific databases encompassing both the technical and healthcare fields. To mitigate selection bias, we implemented a robust study selection process managed independently by two reviewers and verified by a third reviewer. We thoroughly assess the results and identify potential technical challenges faced by AI models in IA recognition tasks. To the best of our knowledge, this is the first review that not only methodologically categorizes but also evaluates the performance of AI models specifically for IA recognition tasks. It integrates the latest trends in AI and medical image analysis, shedding light on technological advancements in the field. Our review summarizes the potential applications of AI in the medical imaging evaluation of intracranial aneurysms, highlighting its valuable role in assisting clinicians with diagnosis and treatment. Furthermore, this review offers researchers and readers insights into the challenges and prospects of developing advanced methods for IA analysis.

### Limitations

The articles selected for this review exhibit varied characteristics such as retrospective methodologies, constrained sample sizes, diverse artificial intelligence algorithms, assorted performance metrics, and distinct methods of outcome measurement. These factors complicate direct comparisons across different models. Furthermore, the limited sample sizes and the absence of standardized metrics have somewhat hindered the broader application and dissemination of these AI models in clinical settings. Despite exhaustive efforts to include research published up to March 2024, we acknowledge the possibility that certain emergent or evolving AI models might not have been captured in this review. Additionally, this analysis is limited to studies published in English, potentially omitting relevant research conducted in other languages.

## Conclusion

This paper aims to conduct a systematic review to understand the current research progress of applying artificial intelligence techniques in intracranial aneurysm recognition. Current studies have achieved good results in IA recognition. But the field still needs more common test benchmarks and public datasets. In addition, the model structure designed by the corresponding research institute is still relatively limited and needs to be improved and enhanced in combination with actual clinical scenarios. In summary, with further improvements, AI methods show favorable prospects in identifying intracranial aneurysms and predicting their potential for rupture and blockage.

## Data Availability

The data used in this study are available on request from the corresponding author.

## Abbreviations

IA: Intracranial aneurysm
AI: Artificial intelligence
SAH: Subarachnoid hemorrhage
CTA: Computed tomography angiograph
MRA: Magnetic resonance angiograph
DSA: Digital subtraction angiograph
ML: Machine learning
DL: Deep learning
CNN: Convolutional neural network
MLP: Multilayer perceptron
SIF: Spatial information fusion
SR: Sparse representation
PCC: Point cloud classification
PCS: Point cloud segmentation
LSTM: Long short-term memory network
SCTR: Staged cluster transformer
MIoU: Mean intersection over union
PA: Pixel accuracy
Sen: Sensitivity
Spe: Specificity
Acc: Accuracy
MIoU: Mean Intersection over Union
IoU: Intersection over Union
EHR: Electronic health record
LLM: Large-language model
AUC: Area Under Curve
